# Ligature-induced periodontitis in mice potentially accelerates CD4^+^ T-cell senescence and exacerbates rheumatoid arthritis

**DOI:** 10.3389/fimmu.2026.1806138

**Published:** 2026-05-26

**Authors:** Jinfeng Li, Terukazu Sanui, Miyu Shida, Karen Yotsumoto, Mwannes Ahmad, Ziyu Wang, Meng Xiao, Chikako Hayashi, Takanori Shinjo, Takaharu Taketomi, Takao Fukuda, Fusanori Nishimura

**Affiliations:** 1 Department of Periodontology, Division of Oral Rehabilitation, Faculty of Dental Science, Kyushu University, Fukuoka, Japan; 2 Dental and Oral Medical Center, Kurume University School of Medicine, Kurume, Fukuoka, Japan

**Keywords:** periodontitis, rheumatoid arthritis, senescence-associated secretory phenotype, senescent CD4+ T cells, thymic involution

## Abstract

**Introduction:**

Aging impairs immunity, sustains chronic inflammation, and enhances autoimmunity–a process termed “immunosenescence” that contributes to the pathogenesis of type 2 diabetes and rheumatoid arthritis (RA). Post-pubertal thymic involution depletes naïve T-cell pool, promoting the emergence of senescent CD4^+^ T cells. To maintain T-cell homeostasis, these cells undergo extensive homeostatic proliferation, eventually reaching their replicative limit. Characterized by PD-1 and CD153 expression, these senescent cells exhibit diminished proliferative capacity and an enhanced senescence-associated secretory phenotype (SASP). While chronic periodontitis, which typically affects middle-aged individuals, is known to influence systemic conditions like RA (periodontal medicine), the underlying mechanisms remain elusive. This study investigates whether periodontitis accelerates CD4^+^ T-cell senescence and its subsequent impact on systemic disease.

**Methods:**

BALB/c mice (5–42 weeks old) underwent silk ligation of the maxillary second molars to induce experimental periodontitis (LIP). Splenic CD4^+^ T cells were isolated and stimulated with IL-2, and anti-TCRβ/CD28 antibodies for 1–3 days to promote T-cell activation and expansion.

**Results:**

Although the frequency of PD-1^+^CD153^+^ cells did not differ significantly between the LIP and control groups *in vivo*, the LIP group showed significantly higher proportion of these double-positive cells following *in vitro* stimulation, peaking at 18 weeks. The LIP group further exhibited elevated SASP cytokine levels, an increased prevalence of senescence-associated β-galactosidase (SA β-gal)-positive cells and a reduced proportion of cells in the S phase, indicating accelerated senescence. RNA-seq analysis revealed numerous differentially expressed genes (DEGs) related to senescence in unstimulated helper T cells from the LIP group. Finally, adoptive transfer of CD4^+^ T cells from the LIP group into nude mice exacerbated collagen antibody-induced arthritis (CAIA).

**Conclusions:**

These findings suggest that severe periodontal inflammation induces a “senescence-primed” status in the helper T cells. These senescent cells may enter systemic circulation and exacerbate RA, highlighting a novel cellular mechanism linking periodontitis to systemic disease.

## 1 Introduction

As medical advances have led to an increasingly super-aged society, concerns arise that the number of diseases driven by the aging of the immune system will increase, and measures to address this are an urgent issue. Generally, immune function gradually declines with age, while chronic inflammation and autoimmune responses increase, leading to an increase in age-related diseases such as metabolic and autoimmune diseases and cancer (1). The decline in immune function due to aging is called “immunosenescence” and has three main characteristics (2). First, a “decline in the adaptive immune response” weakens the resistance to new pathogens. Second, an “increase in proinflammatory traits” makes chronic inflammation more likely to occur. Third, there is an “increased risk of autoimmune diseases” due to an increase in self-reactive cells (3, 4). One of the main causes of the decline in acquired immunity is the rapid shrinkage and decline in function of the thymus gland, which produces T cells that act as immune command centers after puberty. Once adaptive immunity is established, the production of naïve T cells declines owing to thymic involution, and senescent T cells maintain their absolute numbers through “homeostatic proliferation,” which involves proliferating to the limit to compensate for this decline (5, 6). Among T-cell immunosenescence, memory-type helper T cells, termed senescent CD4^+^ T cells (7–9), are characterized by the expression of PD-1, a negative co-stimulatory receptor for T cell receptor (TCR) signaling (10), and CD153, a TNF superfamily protein (11). Senescent CD4^+^ T cells have reduced proliferation after antigen stimulation and shorter telomeres, but exhibit a senescence-associated secretory phenotype (SASP), characterized by the production of large amounts of proinflammatory cytokines (e.g., osteopontin and IL-6) and increased cell-killing capacity (12, 13). In addition, senescent CD4^+^ T cells rapidly increase in number, even in young mice, after thymectomy or T-cell transfer following irradiation (5, 14). Therefore, it is highly likely that this effect was induced by excessive homeostatic proliferation.

Periodontitis is a chronic inflammation in response to bacterial attack that leads to irreversible resorption of the alveolar bone supporting the teeth and ultimately to tooth loss. Chronic periodontitis typically develops around 40 years and is an age-dependent disease rarely observed in individuals under 30 years of age (15, 16). It has been a long time since the concept of “periodontal medicine” first came to the forefront, in which periodontal disease-causing bacteria and their resulting inflammatory products affect organs throughout the body through the bloodstream. For example, numerous reports have shown that periodontal disease affects systemic diseases such as diabetes and rheumatoid arthritis (17–20). Most of these systemic diseases related to periodontal medicine overlap with diseases believed to be caused by T-cell immunosenescence, and the age of onset is roughly the same (21). Recently, a cross-sectional study in the United States found that leukocyte telomere length in patients with periodontitis was significantly shorter than that in age-matched patients without periodontitis. Masi et al. also reported that shortened telomere length was associated with the diagnosis of periodontitis and that telomere length correlated with oxidative stress and the severity of periodontitis (22). These findings suggest a potential association between periodontitis and immunosenescence. However, the cellular mechanisms underlying this association, particularly at the induction of CD4^+^ T-cell senescence, remain to be fully elucidated.

Based on these findings, we hypothesized that chronic periodontitis may initiate and affect senescent CD4^+^ T cells, thereby contributing to systemic disease. In this study, we focused on the immunosenescence of CD4^+^ T cells, which are the command centers of the immune response, and used ligature-induced experimental periodontitis as an experimental model to elucidate the molecular mechanism by which chronic periodontitis exacerbates rheumatoid arthritis, a systemic disease related to periodontal medicine.

## 2 Materials and methods

### 2.1 Animals

Male BALB/c mice (5, 10, 18, 26, 34, and 42-week-old), C57BL/6J (5, 10, 18-week-old) and BALB/c *nu/nu* nude mice (7 weeks old) were purchased from Japan SLC, Inc. (Hamamatsu, Japan). Only male mice were used to minimize sex-dependent immune response effects. BALB/c mice were housed under specific pathogen–free (SPF) conditions with a 12-h light/dark cycle. BALB/c *nu/nu* nude mice were maintained in vinyl isolators under sterile conditions. Mice were provided with standard laboratory chow and water *ad libitum*, undergoing a minimum of seven days of acclimatization prior to the commencement of the study. All animal procedures were approved by the Animal Care and Use Committee of Kyushu University (Permit Number: A25-025-0) and followed institutional and national guidelines.

### 2.2 Ligature-induced periodontitis

Ligature-induced periodontitis was established as previously described (23). Briefly, BALB/c (5–42 weeks) and C57BL/6J mice (5–18 weeks) were anesthetized, and 6–0 silk thread (Akiyama Medical MFG, Tokyo, Japan) was placed bilaterally around the second maxillary molars. Control mice were anesthetized but did not receive ligatures. Fourteen days after ligature placement, mice were euthanized by cervical dislocation while under deep anesthesia induced by an intraperitoneal injection of a tri-drug mixture (0.3 mg/kg medetomidine, 4.0 mg/kg midazolam, and 5.0 mg/kg butorphanol). The depth of anesthesia was confirmed by the loss of the pedal withdrawal reflex prior to euthanasia. Death was confirmed by the cessation of heartbeat and respiration. The maxillae were harvested and fixed in 4% paraformaldehyde for subsequent alveolar bone resorption analysis via micro-computed tomography (micro-CT).

### 2.3 Preparation and culture of splenocytes and CD4^+^ T cell isolation

**Figure 1:**
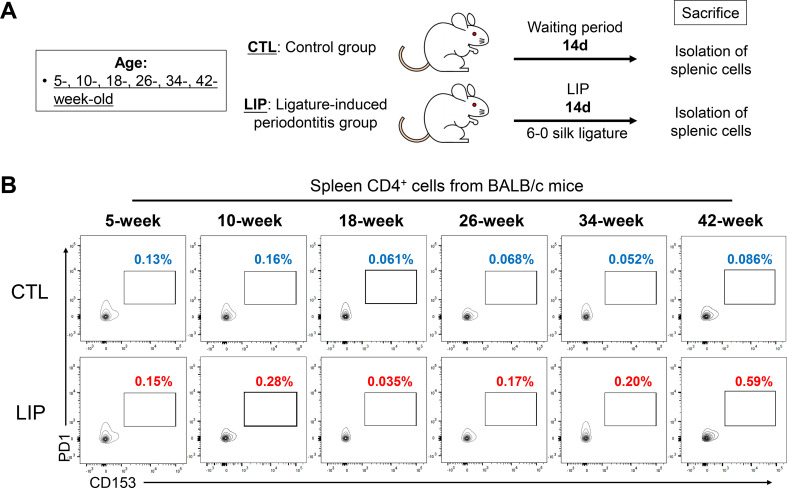
PD-1^+^CD153^+^CD4^+^ T cells were rarely present in the spleen of BALB/c mice of any age. **(A)** Schematic of the experimental design. **(B)** Representative plots display the percentages of PD-1^+^ and CD153^+^ cells in the spleen of the LIP and CTL groups. All plots were gated on live CD4^+^ T cells. Similar results were obtained in five independent experiments.

**Figure 2:**
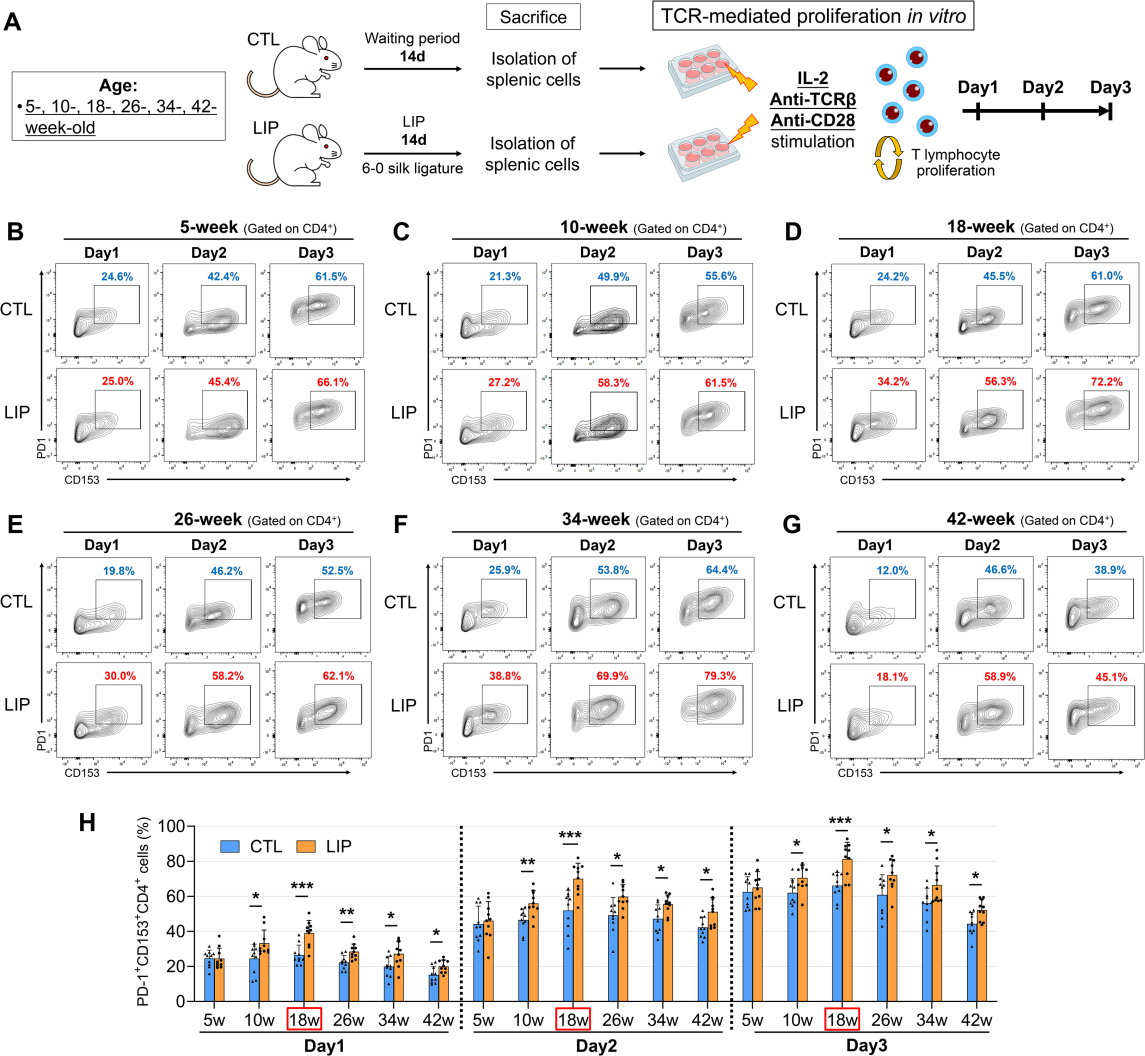
LIP enhances immunosenescence of CD4^+^ T cells by inducing TCR-mediated proliferation *in vitro*. **(A)** Schematic of the experimental design. **(B–G)** Representative plots display the percentages of PD-1^+^ and CD153^+^ cells in the spleen of the LIP and CTL groups at 5 weeks **(B)**, 10 weeks **(C)**, 18 weeks **(D)**, 26 weeks **(E)**, 34 weeks **(F)**, 42 weeks **(G)** of age on days 1, 2, and 3 of *in vitro* culture with IL-2, anti-TCRβ antibody, and anti-CD28 antibody. All plots were gated on live CD4^+^ T cells. **(H)** Quantification of the percentages of PD-1^+^CD153^+^CD4^+^ splenocytes corresponding to the two groups at each age on days 1, 2, and 3 of *in vitro* culture. The significance of differences between groups was determined using a two-tailed unpaired Student’s t-test; *p < 0.05; **p < 0.01; ***p < 0.001. Data represent mean ± SD. Similar results were obtained in ten independent experiments.

**Figure 3:**
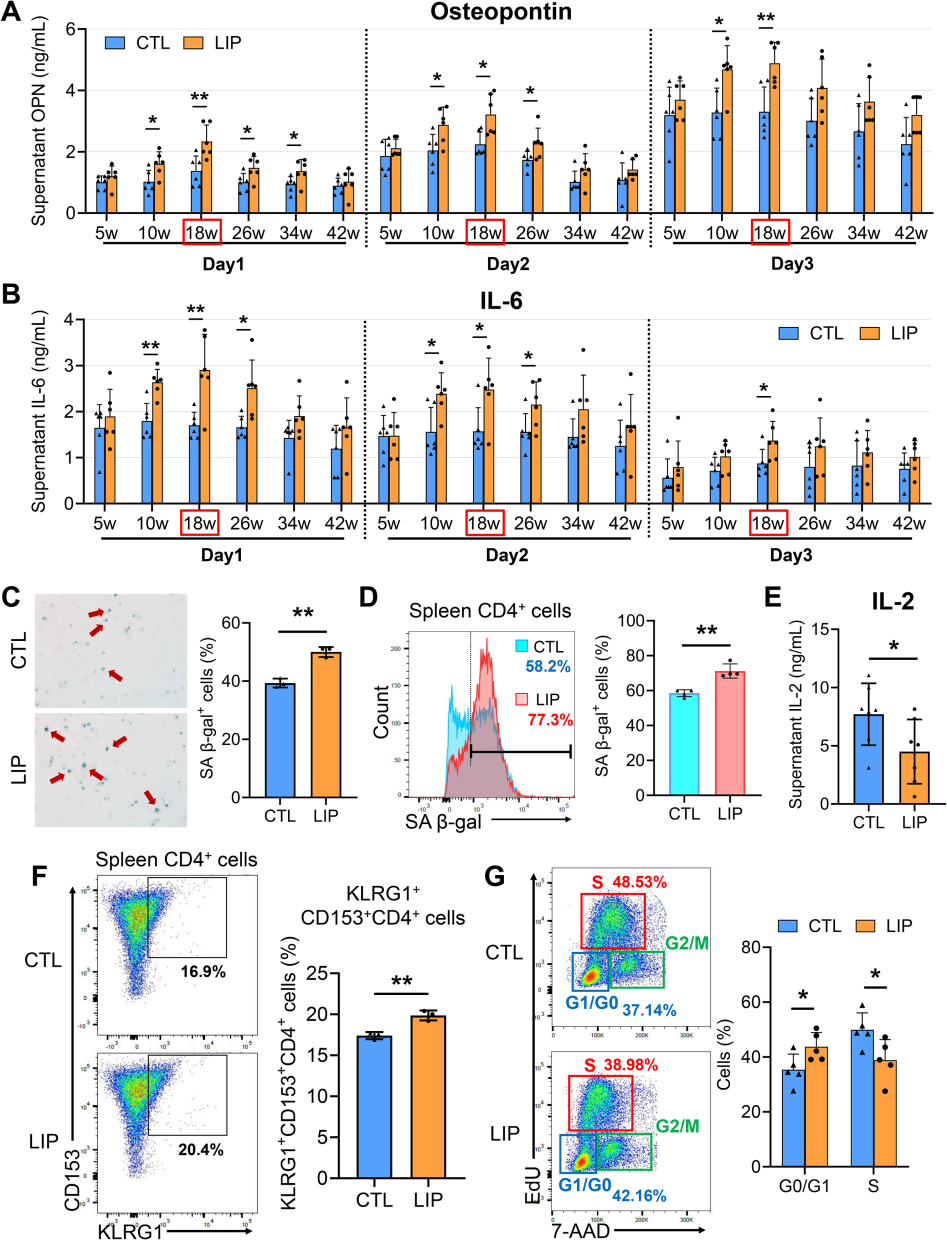
Splenic CD4^+^ T cells in periodontitis-induced mice exhibit immunosenescence characteristics through TCR stimulation. Osteopontin **(A)** and IL-6 **(B)** production by splenic CD4^+^ T cells stimulated with IL-2, anti-TCRβ antibody, and anti-CD28 antibody from 18-week-old mice in the LIP and CTL groups was analyzed in the supernatants on days 1, 2, and 3 (*n* = 6). **(C)** Representative images of senescence-associated β-galactosidase (SA β-gal) staining of the splenic CD4^+^ T cells from 18-week-old mice in the LIP and CTL groups cultured under IL-2, anti-TCRβ antibody, and anti-CD28 antibody for 7 days. Quantification of SA β-gal staining corresponding to the two groups (*n* = 3). Scale bars: 50μm. **(D)** Representative histograms and bar graphs display the percentages of SA β-gal^+^ CD4^+^ cells in the spleen from 18-week-old mice in the LIP and CTL groups on day 2 of *in vitro* culture (*n* = 4). **(E)** Purified splenic CD4^+^ T cells from 18-week-old mice in the LIP and CTL groups were stimulated with *in vitro* for two days. Culture supernatants were collected and analyzed for IL-2 production (*n* = 7). **(F)** Representative blots and bar graphs display the percentage of KLRG1^+^CD153^+^CD4^+^ cells in spleen from 18-week-old mice in the LIP and CTL groups on day 2 of *in vitro* culture (*n* = 3). **(G)** Representative plots and bar graphs of cell cycle analysis display the percentages of G0/G1- and S-phase cells in the spleen from 18-week-old mice in the LIP and CTL groups on day 2 of *in vitro* culture (*n* = 5). The significance of differences between groups was determined using a two-tailed unpaired Student’s t-test; *p < 0.05; **p < 0.01. Data represent mean ± SD.

Spleens were harvested aseptically from mice and mechanically dissociated in RPMI-1640 medium (Nacalai Tesque, Kyoto, Japan) supplemented with 10% heat-inactivated fetal bovine serum (FBS), penicillin, and streptomycin. Red blood cells were lysed using RBC Lysis Buffer (BioLegend, #420302; San Diego, CA, USA). Bulk splenocytes obtained after red blood cell lysis were used either directly or subjected to further purification, depending on the experimental design. Bulk splenocytes were used only for experiments shown in **Figures 1**, **2**, **3A, B**. For experiments requiring purified CD4^+^ T cells, bulk splenocytes were subjected to negative selection using the MojoSort™ Mouse CD4 T Cell Isolation Kit (BioLegend, #480006) according to the manufacturer’s instructions. The purity of isolated CD4^+^ T cells was 95%–98%, as confirmed by flow cytometry.

### 2.4 T cell activation and culture conditions

Six-well plates were precoated overnight at 4 °C with anti-TCRβ antibody (3 µg/mL; BioLegend, #109202) and anti-CD28 antibody (1 µg/mL; BioLegend, #102102) diluted in PBS. Both bulk splenocytes and purified CD4^+^ T cells were stimulated and cultured under identical conditions. Cells were seeded at a density of 1 × 10^6^ cells per well in the precoated plates and cultured in RPMI-1640 medium supplemented with 10% FBS and recombinant mouse IL-2 (2.5 ng/mL; BioLegend, #575406). Cultures were maintained at 37 °C in a humidified incubator with 5% CO_2_ for up to 3 days, with daily medium replacement. For time-course experiments using bulk splenocytes, cells and culture supernatants were collected at the indicated time points (Day 0, Day 1, Day 2, and Day 3) for downstream analyses, including ELISA (**Figures 1**, **2**, **3A, B**). All other experiments were performed using purified CD4^+^ T cells unless otherwise indicated.

Purified CD4^+^ T cells collected immediately after isolation were defined as Day 0, whereas cells collected after two days of stimulation and culture were defined as Day 2.

### 2.5 Flow cytometry

Cells were blocked with Fc Block (BioLegend, #156604) and stained with fluorochrome-conjugated antibodies against CD4-FITC (BioLegend, #103006), PD-1-APC (BioLegend, #135209), CD153-PE (BioLegend, #106405), and killer cell lectin-like receptor G1 (KLRG1)-PerCP (BioLegend, #138417). Fluorescence-minus-one (FMO) and unstained controls were used. Samples were analyzed on a BD FACS Lyric cytometer (BD Biosciences, San Diego, CA, USA) and data processed using FlowJo software 10.10.0 (BD Biosciences).

### 2.6 Micro-computed tomography analysis

Micro-CT scanning of maxillae and the fore limbs was performed using a ScanXmate system (Comscan, Kanagawa, Japan) operated at 50 kV and 160 µA with a voxel size of 11.468 µm. Three-dimensional reconstructions were generated using TRI/3D-BON software (Ratoc System Engineering, Tokyo, Japan). Alveolar bone loss was evaluated by measuring the distance from the cementoenamel junction (CEJ) to the alveolar bone crest (ABC) at five standardized buccal sites per sample. Measurements were performed on reconstructed images using ImageJ software (version 1.53i; National Institutes of Health). The cumulative CEJ–ABC distance was calculated for each specimen. Root surface area was quantified on the same reconstructed images using ImageJ.

### 2.7 Enzyme-linked immunosorbent assay

Osteopontin ([KE10046]; Proteintech, Rosemont, IL, USA), IL-6 (BioLegend, #431301), IL-2 (BioLegend, #431001), and IL-1β (BioLegend, #432604) concentrations in culture supernatants were measured by ELISA per manufacturers’ instructions.

### 2.8 Cell proliferation and senescence assays

EdU incorporation was detected using the Click-iT™ EdU Alexa Fluor™ 488 Flow Cytometry Assay Kit ([C10632]; Thermo Fisher Scientific, Waltham, MA, USA). Senescence-associated β-galactosidase (SA β-gal) activity was evaluated using two complementary approaches. For histochemical detection, SA β-gal activity was assessed using the Senescence β-Galactosidase Staining Kit ([9860S]; Cell Signaling Technology, Danvers, MA, USA) following the manufacturer’s protocol, in which senescent cells were identified by blue staining. Stained images were visualized using a BZ-X800 fluorescence microscope (Keyence, Tokyo, Japan) and analyzed with BZ-X Analyzer software (Keyence). For flow cytometric analysis, intracellular SA β-gal activity was detected using the SPiDER-βGal Cellular Senescence Detection Kit ([SG03]; Dojindo Laboratories, Kumamoto, Japan) according to the manufacturer’s instructions. Samples were analyzed on a BD FACS Lyric cytometer (BD Biosciences), and data were processed using FlowJo software version 10.10.0 (BD Biosciences).

### 2.9 RNA sequencing

Total RNA was extracted from purified CD4^+^ T cells. RNA concentration and integrity were assessed using an Agilent TapeStation system (Software v4.1). Samples with RIN values of 8.3–9.8 were used for library preparation. Sequencing libraries were generated using the MGIEasy RNA Directional Library Prep Set following the manufacturer’s instructions. The libraries were sequenced on the DNBSEQ-G400 platform to generate 150-bp paired-end reads.

### 2.10 Data analysis

Initial quality assessment of raw sequencing data was performed using FastQC (v0.11.9). Low-quality bases and adapter sequences were trimmed using Trimmomatic (v0.36). Cleaned reads were mapped to the reference genome (GRCm39) using HISAT2 (v2.1.0). Gene expression levels were quantified using RSEM (v1.3.0) with Bowtie2. Differential expression analysis was conducted using edgeR, with a significance threshold of *p* < 0.05 to identify DEGs. Adjusted p-values were used in hierarchical clustering heatmap. Gene sets were categorized based on Gene Ontology (GO) terms and Kyoto Encyclopedia of Genes and Genomes (KEGG) pathways. Enrichment analysis was performed using the enrichplot package (v1.16.1) and visualized using ggplot2 (v3.3.6). A protein-protein interaction (PPI) network was constructed using the STRING database (v12.0) and visualized with Cytoscape(v3.10.4).

### 2.11 Quantitative reverse transcription polymerase chain reaction

Total RNA was extracted from CD4^+^ T cells on day 0 and day 2 using Isogen II RNA purification reagent (311-07361; Nippon Gene, Tokyo, Japan). RNA quantity and purity were assessed using a NanoPhotometer N50 Touch (Implen GmbH, Munich, Germany). First-strand cDNA was synthesized using PrimeScript™ RT Master Mix (RR036A; Takara Bio, Kusatsu, Shiga, Japan). qRT-PCR was performed using Luna Universal qPCR Master Mix (M3003; New England BioLabs, Ipswich, MA, USA) on a StepOne Plus™ Real-Time PCR System (Applied Biosystems, Carlsbad, CA, USA). The amplification protocol consisted of an initial denaturation at 95 °C for 3 min, followed by 40 cycles of 95 °C for 3 s and 60 °C for 30 s. Gene expression levels were analyzed using the ΔΔCT method, with 18S rRNA serving as the internal control. Primer sequences used in this study are listed in **Supplementary Table 1**.

### 2.12 Adoptive cell transfer

CD4^+^ T cells from 18-week-old BALB/c mice were activated and cultured under the anti-TCRβ/CD28 stimulation and IL-2-supplemented conditions described above. Activated CD4^+^ T cells (1.2–1.5 × 10^6^ cells per injection) were retro-orbitally injected into BALB/c *nu/nu* mice under light anesthesia every 3 days for a total of four injections. Recipient mice were then maintained for an additional 7 days for *in vivo* expansion and persistence of the transferred cells prior to the induction of arthritis.

### 2.13 Collagen antibody-induced arthritis model

CAIA model was induced in BALB/c *nu/nu* mice seven days after the completion of adoptive T-cell transfer (as described in Section 2.12). The induction was performed using a commercial kit containing an anti-type II collagen antibody cocktail and lipopolysaccharide (LPS) ([53040]; Chondrex, Inc., Woodinville, WA, USA). Briefly, recipient mice received a single intraperitoneal (*i.p.*) injection of 5 mg anti-type II collagen antibody cocktail on day 0, followed by an *i.p*. injection of 50 μg LPS on day 3. Joint swelling (joint swell degree) was assessed daily by counting swollen ankles and toe joints (maximum score = 22). Arthritis severity (arthritis index) was scored on a scale of 0–4 according to the manufacturer’s guidelines.

### 2.14 Histological analysis

After micro-CT imaging, fore limbs were decalcified in 10% EDTA, paraffin-embedded, sectioned at 5 µm, and stained with hematoxylin and eosin (H&E) for bone assessment. Stained images were visualized using a BZ-X800 fluorescence microscope (Keyence). Images were analyzed with BZ-X Analyzer software (Keyence). For immunofluorescence staining, non-specific staining was blocked with G-Block ([GB-01]; Genostaff, Tokyo, Japan) for 30 min at room temperature. The sections were then incubated overnight at 4 °C in the dark with the following primary antibodies: anti-CD4 antibody ([GK1.5]; Thermo Fisher Scientific), anti-CD153 antibody ([C10540]; Assay Biotechnology Company, Inc., San Jose, CA, USA). After washing, the slides were incubated with appropriate secondary antibodies. Nuclei were counterstained and mounted using SlowFade™ Diamond Antifade Mountant with DAPI (Thermo Fischer Scientific). Images were acquired using a confocal laser scanning microscope (LSM700, Carl Zeiss, Oberkochen, Germany) and ZEN 2012 software (Carl Zeiss). The number of CD153^+^CD4^+^ double-positive cells per field was quantified using ImageJ software (National Institute of Health, Bethesda, MD, USA).

### 2.15 Statistical analysis

All data are expressed as mean ± SD. Differences between the two groups were analyzed using Student’s t-test. Statistical significance was set at *p* value < 0.05. All statistical analyses were performed using GraphPad Prism version 10.4.2. (GraphPad Software Inc., La Jolla, CA, USA).

## 3 Results

### 3.1 PD-1^+^CD153^+^CD4^+^ T cells were rarely present in the spleen of BALB/c mice of any age

To investigate whether the proportion of senescent CD4^+^ cells correlated with mouse age and whether there were differences in the number of senescent CD4^+^ cells between the periodontitis model and control groups, we analyzed the presence of PD-1^+^CD153^+^CD4^+^ cells using flow cytometry. To induce experimental periodontitis, BALB/c mice at 5, 10, 18, 26, 34, and 42 weeks of age were placed with a 6–0 silk ligature around the maxillary second molar (LIP group) or not (CTL group). After 14 days, the mice from both groups were sacrificed, their spleens were removed, and splenocytes were isolated (**Figure 1A**). The results showed that CD4^+^ T cells coexpressing PD-1 and CD153 from BALB/c mice were rarely detected in either group (**Figure 1B**) across the six age groups. In contrast, while PD-1^+^CD153^+^CD4^+^ cells were virtually absent in the spleens of C57BL/6J mice aged 5 to 18 weeks, the proportion of PD-1^+^CD153^−^CD4^+^ cells increased slightly in an age-dependent manner (**Supplementary Figure 1**). Previous studies have reported that splenic PD-1^+^CD153^+^CD4^+^ cells in C57BL/6J mice emerge after 50 weeks of age, suggesting that CD153 expression may become more prominent during the later stages following PD-1 induction (24–26); thus, the co-expression of PD-1 and CD153 is considered a reliable indicator of the senescent phenotype. The accumulation of PD-1^+^CD153^+^ cells may be attributed to the intrinsic Th1/Th2 immune bias determined by the genetic background. C57BL/6J mice are characterized by robust Th1-mediated cellular immune responses and active production of IFN-γ, which may trigger the upregulation of inhibitory molecules such as PD-1 as a feedback mechanism to modulate intense immune activation (27, 28). Furthermore, given that the deficiency of CD153 signaling in the C57BL/6J strain suppresses age-related changes and inflammatory dysfunction (26, 29), CD153^+^ cells may preferentially differentiate and accumulate under chronic antigen exposure or Th1-skewed inflammatory environments. Consequently, the increase in CD153 positivity in response to aging or stimuli may be particularly pronounced in this strain.

### 3.2 LIP enhances immunosenescence of CD4^+^ T cells by inducing TCR-mediated proliferation *in vitro*

Spleen cells isolated from mice in the LIP and CTL groups at each age were stimulated to proliferate with recombinant IL-2, anti-TCRβ, and anti-CD28 antibodies (30). PD-1 and CD153 double-positive cells were analyzed by flow cytometry on days 1, 2, and 3 of *in vitro* culture (**Figure 2A**). In 5-week-old mice, the proportion of double-positive cells gradually increased daily over the course of culture; however, no significant difference was observed between the two groups (**Figures 2B, H**). In 10-week-old mice, the proportion of double-positive cells further increased and was significantly higher in the LIP group than in the CTL group (**Figures 2C, H**). At 18 weeks of age, the proportion of PD-1^+^CD153^+^CD4^+^ cells in the LIP group peaked, and the most significant difference was observed on days 1–3 compared to that in the control group (**Figures 2D, H**, red box). At 26 weeks of age, the proportion of PD-1^+^CD153^+^CD4^+^ T cells began to decrease, but significant differences remained between the two groups (**Figures 2E, H**). A similar trend was observed in 34- and 42-week-old mice (**Figures 2F–H**). These findings suggest that CD4^+^ T cells that undergo ligature-induced periodontitis exhibit an increased propensity to acquire senescence-associated phenotypes upon TCR-mediated proliferative stimulation, with this effect peaking at 18 weeks of age.

### 3.3 Alveolar bone resorption due to experimental ligature-induced periodontitis peaks at 18 weeks of age

**Figure 4:**
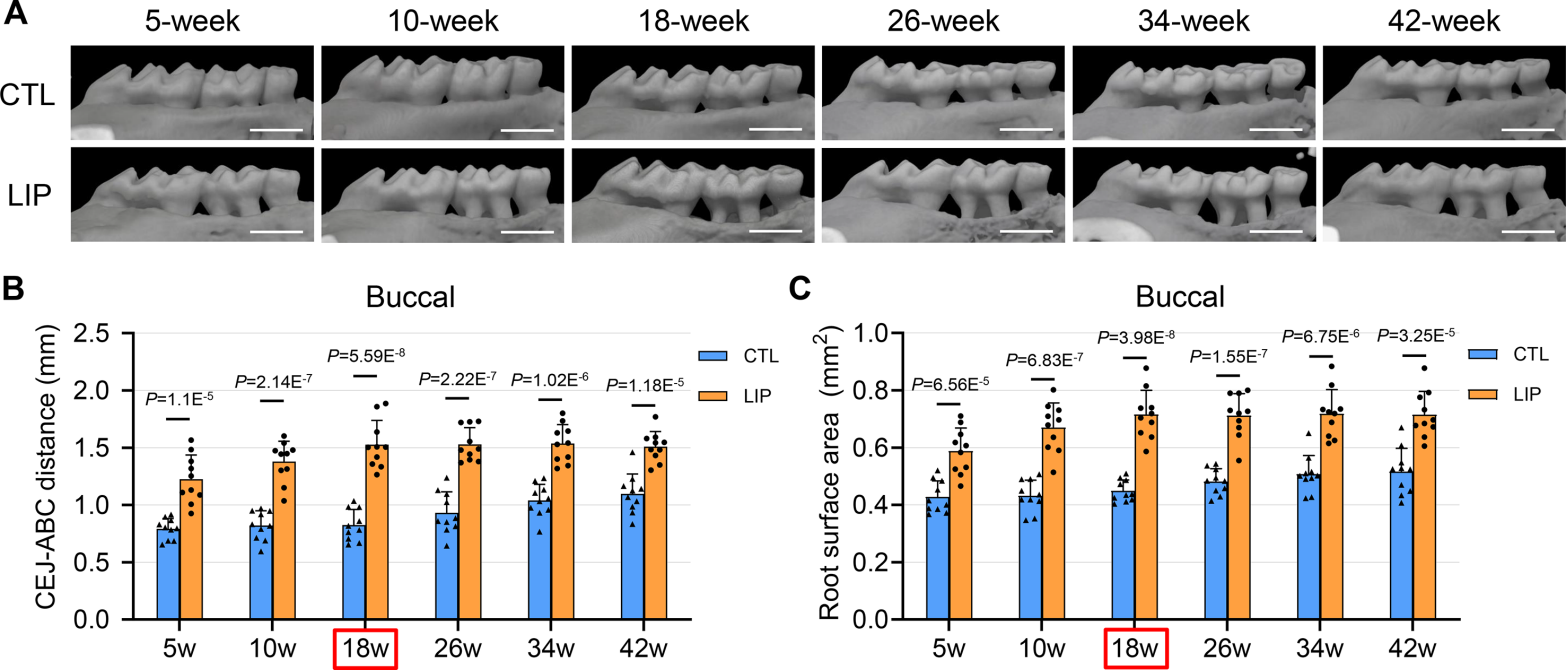
Alveolar bone resorption due to experimental ligature-induced periodontitis peaks at 18 weeks of age. **(A)** Three-dimensional micro-CT, micro-computed tomography images of the maxillae in the LIP and CTL groups at each age. Scale bars: 1,000 μm. **(B)** Quantified buccal alveolar bone loss based on the distances between CEJ, cemento–enamel junction and the ABC, alveolar bone crest of each in mice of different age with or without ligature for 14 days. **(C)** Quantified alveolar bone loss based on buccal root surface area of maxillary molars in mice of each age with or without ligature for 14 days. The significance of differences between groups was determined using a two-tailed unpaired Student’s t-test. Data represent mean ± SD. Similar results were obtained in ten independent experiments.

To investigate why TCR stimulation-mediated immunosenescence was most strongly induced in the LIP group of 18-week-old mice, we analyzed the alveolar bone resorption caused by periodontitis at each age using micro-CT. LIP-induced alveolar bone resorption, measured as the distance from the cemento-enamel junction (CEJ) to the alveolar bone crest (ABC) (**Figures 4A, B**) or root surface area (**Figures 4A, C**), was observed in mice at 5 weeks of age, peaked at 18 weeks of age, and plateaued thereafter. In contrast, no significant changes in alveolar bone resorption were observed in the CTL group from 5 to 18 weeks of age. However, spontaneous alveolar bone resorption occurred from 26 weeks of age onwards, despite the absence of ligation (**Figures 4A–C**). The most significant difference in bone resorption between the CTL and LIP groups occurred in 18-week-old mice (CEJ-ABC distance; 0.82 ± 0.14 mm versus 1.53 ± 0.21 mm, *p* = 5.59E^-8^, *n* = 10 mice/group) (**Figure 4B**), similar to the detection of senescent CD4^+^ T cells in **Figure 2**. This finding suggests that alveolar bone resorption due to periodontitis does not increase with age in mice and that there is a correlation between the progression of periodontal inflammation and the accelerated immunosenescence of helper T cells.

### 3.4 Splenic CD4^+^ T cells in periodontitis-induced mice exhibit immunosenescence characteristics through TCR stimulation

As shown in **Figure 2**, ligature-induced periodontitis increased the proportion of splenic PD-1^+^CD153^+^CD4^+^ cells via TCR stimulation. Therefore, we investigated whether these cell populations exhibited the characteristics of senescent CD4^+^ T cells. The SASP phenotype is characterized by the production of proinflammatory factors such as IL-6 and osteopontin (OPN) (31–33). As shown in **Figure 2**, splenocytes were isolated from mice of various ages and subjected to *in vitro* proliferative stimulation. The concentrations of OPN (**Figure 3A**) and IL-6 (**Figure 3B**) in the culture supernatants on days 1, 2, and 3 of culture were measured using ELISA. Stimulation with a combination of IL-2, anti-TCRβ antibody, and anti-CD28 antibody enhanced OPN production in the culture supernatants of LIP mice, peaking at 18 weeks of age (Day 3; 3.31 ± 0.81 ng/mL versus 4.88 ± 0.68 ng/mL, **p = 0.00448, *n* = 6 mice/group) (**Figure 3A**). Similar to the results for PD-1/CD153 double-positive mice, the greatest significant difference in IL-6 levels was observed in 18-week-old mice throughout days 1–3 of culture (Day 1; 1.79 ± 0.39 ng/mL versus 2.64 ± 0.28 ng/mL, **p = 0.00158, *n* = 6 mice/group) (**Figure 3B**). A 2-day culture of splenocytes from 18-week-old mice resulted in an increased proportion of senescent CD4^+^ T cells and stable SASP secretion. Under these conditions, we measured the activity of senescence-associated β-galactosidase (SA β-gal), a marker of cellular senescence, between the LIP and CTL groups. Compared with the CTL group, the percentage of SA β-gal-positive cells in the LIP group increased 2 days after TCR stimulation (**Figure 3C**). Flow cytometry analysis also detected approximately 1.3-fold stronger expression of SA β-gal in CD4^+^ T cells from the LIP group compared with the CTL group (**Figure 3D**). It is well established that IL-2 production in T cells increases upon activation but declines with senescence. This reduced production is a hallmark of the T-cell senescence phenotype (34). To determine whether *in vitro*-stimulated PD-1^+^CD153^+^CD4^+^ T cells exhibit inherent senescence, we assessed their capacity for IL-2 production. Analysis of purified CD4^+^ T cells from the spleens of 18-week-old mice revealed a significant reduction in IL-2 production capacity within the LIP group (**Figure 3E**). Identifying senescent T cells requires a nuanced approach to distinguish them from overlapping phenotypes, particularly, exhausted T cells (35). While persistent high expression of PD-1 is a hallmark of T-cell exhaustion, it is also frequently observed in senescent T cells. Furthermore, senescent cells are characterized by the increased expression of killer cell lectin-like receptor G1 (KLRG1) in addition to CD153. Notably, given the reported negative correlation between PD-1 and KLRG1 expression (36), we employed flow cytometry to analyze KLRG1 expression specifically within the CD153^+^CD4^+^ T-cell population. Our results demonstrated that proliferative stimulation significantly increased the frequency of KLRG1^+^CD153^+^CD4^+^ T cells in the LIP group compared to the control group (**Figure 3F**). Next, to assess the effect of LIP on the cell cycle, we measured the percentage of replicating cells after incubation with 5-ethynyl-2’-deoxyuridine (EdU). Approximately 50% of CD4^+^ T cells in the CTL group were EdU-positive, indicating replication (S phase), whereas the EdU-positive rate of CD4^+^ T cells in the LIP group was reduced to approximately 40% (**Figure 3G**). Taken together, these results indicate that CD4^+^ T cells from LIP mice exhibit multiple hallmarks of cellular senescence upon TCR-mediated proliferative stimulation. These features include not only the co-expression of PD-1 and CD153 but also enhanced SASP production, increased SA β-gal activity, and impaired proliferative capacity.

### 3.5 Effect of LIP on the transcriptional profile of splenic CD4^+^ T cells

**Figure 5:**
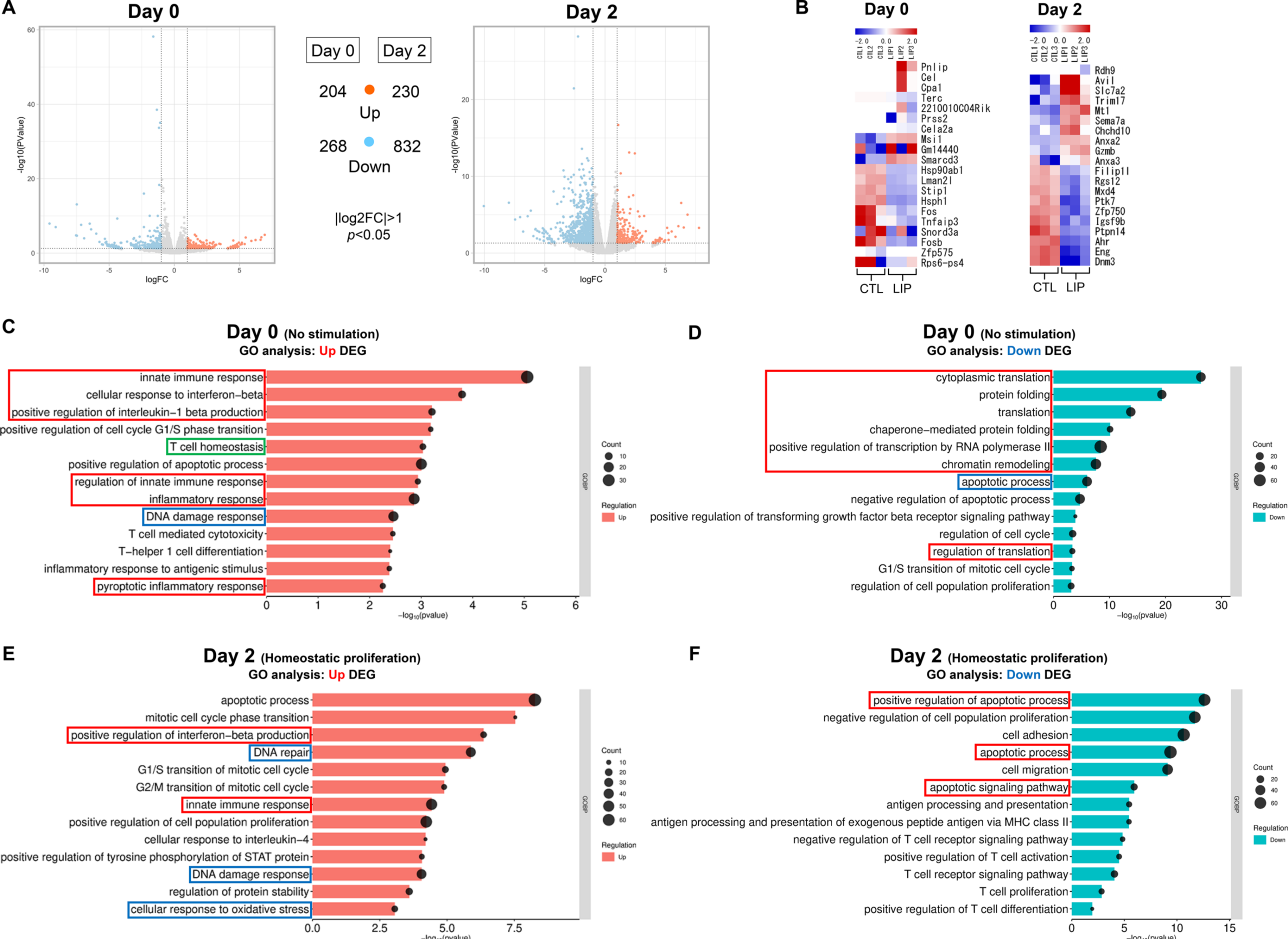
Impacts of LIP on the transcription profile on senescent CD4^+^ T cells. **(A)** Volcano plots displaying DEGs, differentially expressed genes on Day 0 (left) and Day 2 (right). Orange and light blue dots represent upregulated and downregulated transcripts, respectively (|log_2_FC|>1, *p* < 0.05). **(B)** Hierarchical clustering heatmap of the top DEGs (*n* = 3) in each group. Adjusted p-values were used. GO and KEGG analysis of the role of upregulated DEGs and screening enrichment pathway on day 0 **(C)** or day 2 **(E)** after stimulation with IL-2, anti-TCRβ antibody, and anti-CD28 antibody. GO and KEGG analysis of the role of downregulated DEGs and screening enrichment pathway on day 0 **(D)** or day 2 **(F)** after stimulation. Select the 10 most significant KEGG pathways to draw a scatter diagram for display. The length of the bars represents the significance levels (-log_10_ *p* value), and the dot size indicates the gene count associated with each GO term.

To investigate the potential molecular mechanisms underlying the induction of senescent CD4^+^ T cells by LIP, we performed RNA sequencing (RNA-seq) of splenic CD4^+^ T cells from the LIP and CTL groups on day 0 without stimulation and on day 2 after TCR-mediated activation. Compared with the CTL group, 472 differentially expressed genes (DEGs), including 204 upregulated and 268 downregulated genes, were detected in the LIP group on day 0, and 1,062 DEGs, including 230 upregulated and 832 downregulated genes, were detected on day 2. A more extensive and significant response was observed on day 2 than on day 0, particularly for downregulated genes (**Figure 5A**). A heatmap of the top ten genes is shown in **Figure 5B**. Gene Ontology (GO) and Kyoto Encyclopedia of Genes and Genomes (KEGG) analysis of the upregulated genes revealed that the DEGs upregulated on day 0 were related to innate immune inflammatory responses, such as “innate immune response,” “cellular response to interferon β (IFNβ),” and “positive regulation of interleukin-1β (IL-1β) production” (red box). Furthermore, the DEGs included terms related to cellular senescence, such as “T-cell homeostasis” (green box), “DNA damage response,” (blue box) and “pyroptosis inflammatory response” (**Figure 5C**). Meanwhile, the downregulated DEGs on day 0 were suppressed in “chromatin remodeling,” “transcription,” “translation,” and “protein folding,” as shown in red boxes, highlighting the so-called “loss of proteostasis” (**Figure 5D**). The upregulated DEGs on day 2 were involved in “positive regulation of IFNβ” and “innate immune response,” as on day 0 (red box), but also involved “DNA repair,” “DNA damage response,” and “cellular response to oxidative stress” (blue box) (**Figure 5E**). The downregulated DEGs on day 2 included terms related to “apoptotic process” and “apoptotic signaling pathway” (red box), which may suggest apoptosis resistance characteristic of senescent cells (**Figure 5F**).

**Figure 6:**
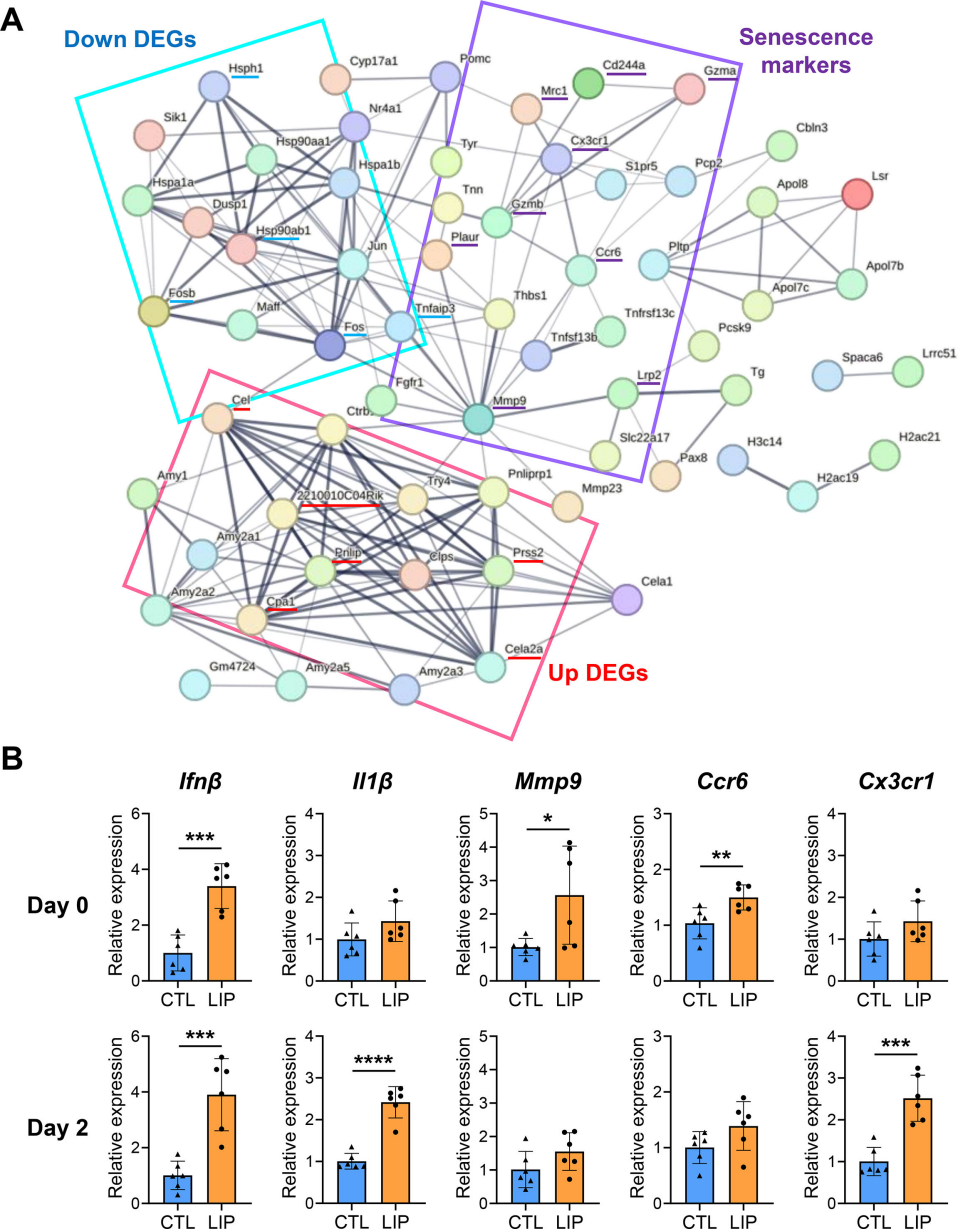
LIP potentially accelerates senescence in CD4^+^ T cells at the genetic level. **(A)** PPI analysis and screening of the hub gene and key signaling pathways in DEGs on day 0. Red and blue clusters indicate upregulated and downregulated genes, respectively. The purple cluster indicates the *matrix metalloproteinase 9* (*Mmp9*)-associated module containing senescence-related genes. **(B)** Splenic CD4^+^ T cells from 18-week-old mice in the LIP and CTL groups cultured under IL-2, anti-TCRβ antibody, and anti-CD28 antibody for 0 or 2 days were subjected to total RNA extraction, followed by the determination of mRNA expression corresponding to *Ifnβ, interferon β*; *Il1β, interleukin-1β*, *Mmp9*; *Ccr6, C-C motif chemokine receptor 6*; and *Cx3cr1, C-X3-C motif chemokine receptor 1* with qRT-PCR (*n* = 6). The significance of differences between groups was determined using a two-tailed unpaired Student’s t-test; *p < 0.05; **p < 0.01; ***p < 0.001; ****p < 0.0001. Data represent mean ± SD.

Next, protein-protein interaction (PPI) analysis was performed based on the RNA-seq results, and three modules were identified on day 0 (**Figure 6A**). The hub gene group, surrounded by red, primarily contains upregulated genes, whereas the module surrounded by blue contains numerous downregulated genes. The hub gene that strongly linked the red and blue boxes was *matrix metalloproteinase 9* (*Mmp9*), a major SASP (37, 38). Senescence-related genes such as *motif chemokine receptor 6* (*Ccr6*) (39) and *C-X3-C motif chemokine receptor 1* (*Cx3cr1*) (40) were identified in the module surrounded by purple, which was derived from *Mmp9*. On the other hand, a large number of genes were detected on Day 2 and identified a module exhibiting senescence, similar to that observed in unstimulated T cells. Other functional clusters closely related to senescence, such as “SASP,” “stress response,” “mitochondrial respiration,” and “lipid metabolism,” were also identified on Day 2 (**Supplementary Figure 2**). Quantitative reverse transcription PCR (qRT-PCR) was used to verify whether LIP affected the expression of these senescence-related hub genes. Compared to the CTL group, the expression of hub genes, including *Mmp9* and *Ccr6* was significantly increased in unstimulated CD4^+^ T cells from the LIP group, whereas the expression of the *Cx3cr1* gene in the LIP group was significantly enhanced on day 2 after TCR stimulation (**Figure 6B**). *Ifnβ* and *Il1β* are related to the “innate immune response,” in which the GO analysis in **Figures 5C, E** showed upregulated DEGs. Although there was no difference in *Il1β* between the LIP and CTL groups on day 0, the expression of both increased in the LIP group on day 2 (**Figure 6B**). These findings suggest that LIP primes CD4^+^ T cells at the transcriptional level, rendering them more susceptible to senescence-associated changes upon subsequent stimulation.

### 3.6 Splenic senescent CD4^+^ T cells from the LIP group exacerbate a collagen antibody-induced arthritis in nude mice

**Figure 7:**
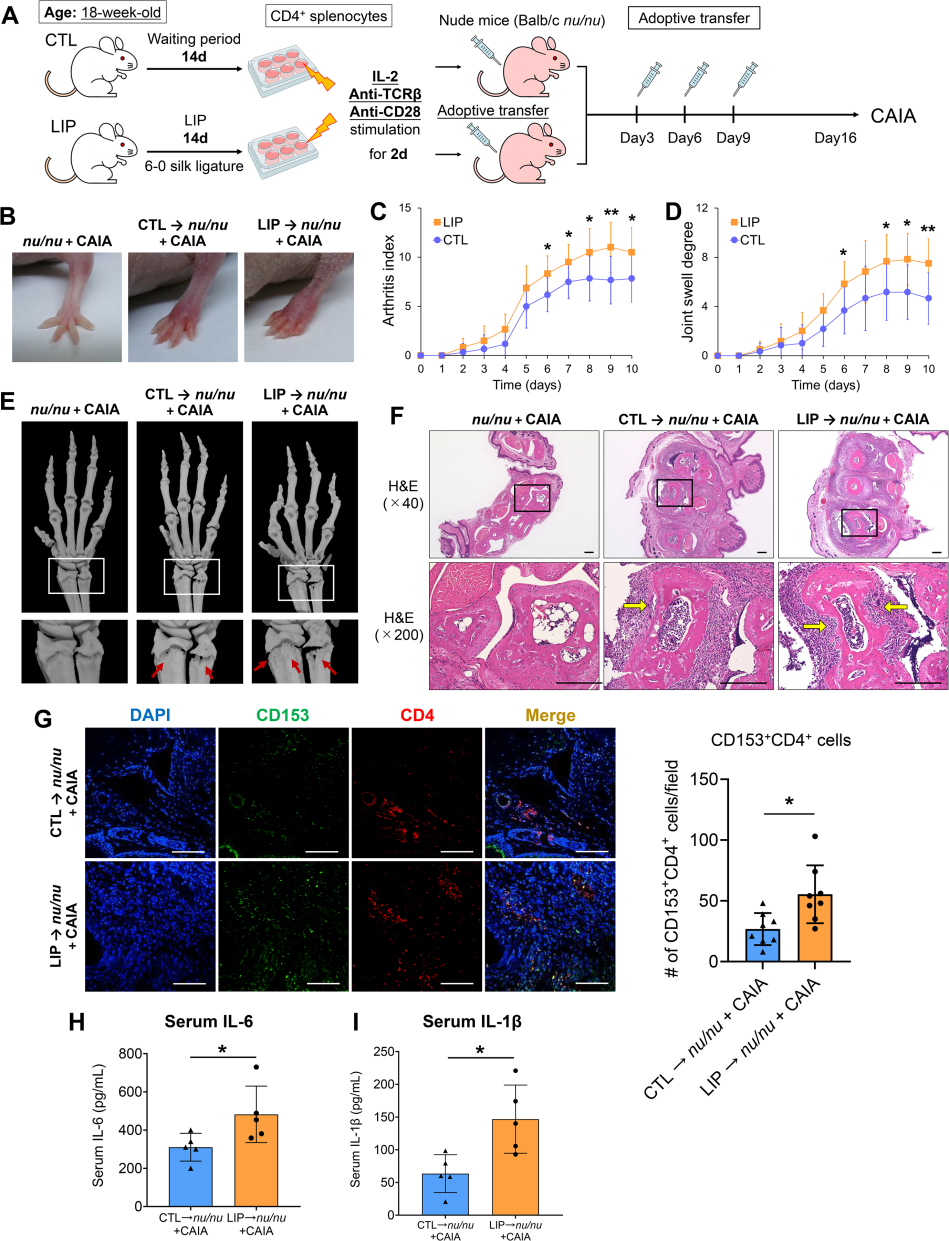
Splenic senescent CD4^+^ T cells from the LIP group exacerbate a CAIA, collagen antibody-induced arthritis in nude mice. **(A)** Schematic of the experimental design. **(B)** The photographs of fore limbs have been shown in nude mouse CAIA model (left panel), and nude mouse CAIA model injected with CD4^+^ T cells from the CTL group (middle panel), or from the LIP group (right panel). **(C, D)** Statistical analysis of arthritis index (*n* = 6) **(C)** and joint swell degree (*n* = 6) **(D)** levels of limbs from day 0 to 10 after CAIA induction. Score levels indicate arthritis index and joint swell degree in nude mouse CAIA model injected with CD4^+^ T cells from the LIP or CTL groups. **(E)** Representative three-dimensional micro-CT images of the fore limbs from each nude mouse CAIA model. Arrows indicate bone destruction. **(F)** Representative images of H&E staining of the fore limbs from each nude mouse CAIA model. Arrows indicate bone destruction. Scale bars: 200 μm. H&E, hematoxylin and eosin. **(G)** Representative immunofluorescence images of forelimb sections from the nude mouse CAIA model following the adoptive transfer of CD4^+^ T cells from the CTL or LIP groups. Sections were stained for DAPI (blue), CD153 (green), and CD4 (red). The right panel shows the quantification of CD153^+^CD4^+^ double-positive cells (yellow) per field for both groups (*n* = 8). Scale bar: 100 μm. **(H, I)** Serum levels of IL-6 **(H)** and IL-1β **(I)** were measured by ELISA in the same CAIA models. Peripheral blood was collected post-adoptive transfer (*n* = 5). The significance of differences between groups was determined using a two-tailed unpaired Student’s t-test; *p < 0.05; **p < 0.01. Data represent mean ± SD.

Because LIP actively altered the expression of various genes in CD4^+^ T cells, we used a collagen antibody-induced arthritis (CAIA) experimental model to examine how LIP-induced senescent CD4^+^ T cells affect rheumatoid arthritis. Nude mice lack a thymus and therefore do not develop CAIA because of the absence of functional T lymphocytes. The spleens removed from the LIP and CTL groups of 18-week-old mice were purified into CD4-positive cells and were adoptively transplanted into nude mice after two days of proliferative stimulation. Senescent CD4^+^ T cells were repeatedly injected into nude mice on days 3, 6, and 9 after the initial transplantation, and after a 1-week interval, CAIA was induced by administering an arthritogenic monoclonal antibody cocktail and lipopolysaccharide (LPS) (**Figures 7A, B**). The limbs of the mice were observed daily to evaluate the clinical scores of arthritis and ankle swelling, and the right fore limbs were harvested for histological examination 10 days after CAIA induction. The clinical arthritis index (severity and intensity of inflammatory changes; **Figure 7C**) and degree of joint swelling (extent and distribution of joint damage; **Figure 7D**) were significantly increased in nude mice transplanted with senescent CD4^+^ T cells from the LIP group compared to those from the CTL group. Micro-CT imaging and H&E staining showed that the joint bone and cartilage destruction increased in the LIP group (**Figures 7E, F**). To investigate the mechanism by which LIP exacerbates rheumatoid arthritis, we performed immunofluorescence staining of CD4^+^ T cells infiltrating the joints. The results showed that CD153^+^CD4^+^ T cells from the LIP group exhibited greater accumulation in the joints of CAIA-induced nude mice compared to those from the CTL group (**Figure 7G**). Furthermore, serum analysis of these nude mice revealed significantly elevated concentrations of IL-6 and IL-1β in the group transplanted with LIP-derived CD4^+^ T cells (**Figure 7H**). These findings suggest that senescent helper T cells, modulated by periodontitis, migrate to the joints and drive the progression of rheumatoid arthritis, subsequently triggering the systemic release of inflammatory cytokines into the bloodstream.

## 4 Discussion

How does LIP, a local inflammation, induce senescence in CD4^+^ T cells? Periodontal pathogens possess virulence factors that can directly induce cellular senescence by releasing bacterial genotoxins, such as cytolethal distending toxin (CDT), or indirectly by using LPS as an antigen. Recently, it was reported that LPS from the periodontal pathogen *Porphyromonas gingivalis* (*P. gingivalis*) can induce premature senescence in alveolar bone cells, which is associated with SASP, which may further promote alveolar bone resorption (41). Similarly, *P. gingivalis* can promote immunosenescence in dendritic cells through direct tissue infiltration, and P. gingivalis-induced senescent dendritic cells may acquire a SASP characterized by the secretion of exosomes loaded with inflammasome-related cytokines and aging-related micro RNAs (42, 43). Pathological changes in tissues affected by periodontitis may create a favorable environment for the induction of cellular senescence, a mechanism that promotes cell survival at the expense of function and often involves the proinflammatory secretome, which may contribute to the maintenance of chronic inflammation. However, it remains unclear whether periodontal pathogens and their virulence factors induce senescence in the T lymphocytes that infiltrate periodontal tissues.

Even when periodontitis was induced by tooth ligation, CD4^+^ T cells in the spleen did not express senescence markers in the mice of any age group (**Figure 1**). However, RNA-seq analysis revealed an elevated expression of genes related to innate immune inflammatory responses in unstimulated helper T cells (**Figure 5C**). Innate immunity and cellular senescence are closely related, and cellular senescence activates innate immune signaling pathways. Conversely, innate immune responses (especially inflammatory cytokines such as type I interferon and IL-1β) can also induce cellular senescence. This positive feedback loop perpetuates chronic inflammation, leading to tissue damage and disease progression (44, 45). In particular, IFNβ, a representative type I interferon, is produced in response to stress signals such as DNA damage and functions as an important mediator of cellular senescence (46). IFN-β, along with IL-1β, is an important component of the SASP and also plays a role in promoting the recognition and elimination of senescent cells by innate immunity (47). Furthermore, as shown in **Figure 5D**, unstimulated CD4^+^ T cells in the LIP group showed a progression of “loss of proteostasis,” a hallmark of aging (48, 49). The results of the PPI analysis revealed that in the vicinity of the upregulated and down-regulated gene groups, in addition to *Cx3cr1* and *Ccr6*, the module with *Mmp9* as the hub gene also contained biomarkers of senescent cells, such as *Plaur* (50), *Gzma* (51), *Gzmb* (52, 53), and *Cd244a* (54, 55).

In this study, latent cellular senescence in the LIP group was further revealed by TCR-mediated activation, and these cells aggravated rheumatoid arthritis in adoptively transplanted nude mice (**Figure 7**). Regarding the relationship between periodontitis and rheumatoid arthritis, a mechanism has been proposed in which proteins in periodontal tissues are citrullinated by a citrullinating enzyme produced by *P. gingivalis*, which produces anti-citrullinated protein antibodies. These antibodies form immune complexes with citrullinated proteins in the joints, leading to arthritis (56, 57). In this study, CD4^+^ T cells affected by periodontitis were adoptively transferred into nude mice, demonstrating that periodontitis exacerbates rheumatoid arthritis through a pathway separate from that involving citrullinated proteins, which is different from the previously proposed mechanism by which periodontal disease worsens rheumatoid arthritis. Cellular senescence is one of the underlying factors that causes persistent inflammation and tissue destruction in rheumatoid arthritis. For example, fibroblast-like synoviocytes (FLS) in the synovial membrane of patients with rheumatoid arthritis undergo senescence and release SASP factors, which induce inflammation in surrounding healthy cells and exacerbate and sustain arthritis (58, 59). It has also been reported that the level of senescent CD4^+^ T cells is higher in patients than in healthy individuals (60–62). These findings indicated a close causal relationship between periodontitis, senescence, and rheumatoid arthritis.

For periodontitis to exacerbate systemic inflammation via immunosenescence, the systemic circulation of senescent immune cells is essential. Two primary pathways are hypothesized to drive this mechanism: first, chronic periodontitis generates inflammatory mediators, bacterial components, or damage-associated molecular patterns (DAMPs) that enter the bloodstream and modulate immune cell functions in secondary lymphoid tissues; second, CD4^+^ T cells undergo senescence directly within inflamed periodontal tissues and subsequently enter the circulation. However, based on the findings of this study, we propose an alternative hypothesized mechanism: periodontal inflammation does not immediately generate a large pool of circulating senescent T cells. Instead, it induces a “pre-senescent” or “senescence-primed” state in the CD4^+^ T cells within the periodontal environment, rendering them highly susceptible to aging upon subsequent stimulation. Once these primed CD4^+^ T cells circulate and encounter pre-existing systemic inflammatory conditions, they undergo rapid senescence driven by proliferative exhaustion. This, in turn, exacerbates systemic diseases through the production of SASP. This interpretation is supported by our transcriptome analysis, which revealed significant transcriptional alterations in innate immune responses, proteostasis, and cellular stress pathways. Such genetic modifications may represent early immunological imprinting that enhances the senescence susceptibility of helper T cells.

This study had three major limitations. First, the LIP model is a powerful tool for investigating rapid, bacteria-mediated bone resorption. Compared to models involving the direct application of periodontal pathogens, the LIP model offers superior control over the extent of bone loss and the onset of inflammation, ensuring high reproducibility. However, the LIP model is inherently invasive and does not fully recapitulate the chronic, slow progression characteristic of human periodontitis. It is therefore necessary to consider whether such acute inflammatory insults might exert excessive stress, potentially inducing an artificial “aged” phenotype. In the present study, however, splenic CD4^+^ T cells from the LIP group did not exhibit a senescent phenotype (**Figure 1B**). These findings suggest that LIP-induced inflammation does not directly trigger systemic T-cell senescence through acute inflammatory toxicity; rather, it may prime CD4^+^ T cells, rendering them susceptible to subsequent aging. Second, it remains unclear whether TCR stimulation using IL-2, anti-TCRβ antibodies, and anti-CD28 antibodies *in vitro* reflects the physiological induction of homeostatic proliferation and subsequent senescence of CD4^+^ T cells *in vivo* although previous studies have shown that inducing T-cell senescence *in vitro* using the same antibody cocktail is effective (30). This *in vitro* stimulation is a well-established method for inducing robust T-cell activation, rapid proliferation, and differentiation (63, 64). However, such intensive stimulation may be more representative of antigen-driven activation than homeostatic proliferation, given that the latter is typically mediated by self-peptide-major histocompatibility complex (MHC) (‘tonic’ signaling) and increased IL-7 availability in lymphopenic hosts (65). Therefore, even if T cells exhibit senescence-like phenotypes following TCRβ/CD28/IL-2 stimulation, this does not necessarily imply a direct link to homeostatic proliferation *per se*. Our findings should therefore be interpreted in the context of activation-induced proliferative stress rather than bona fide homeostatic proliferation. Furthermore, the precise time course required for homeostatic proliferation to occur *in vivo* remains to be elucidated. Future studies should focus on developing a method to induce immunosenescent cells *in vivo* by transferring helper T cells isolated from tooth-ligated mice into athymic mice or lymphopenic mice without the need for *in vitro* stimulatory expansion. Third, the higher proportion of senescent CD4^+^ T cells in the LIP group does not necessarily explain the stronger inflammatory response in the joints compared to the CTL group in cell transplantation and CAIA experiments in nude mice. In other words, helper T cells sensitized by periodontal bacteria may differentiate into memory effector T cells regardless of senescence, contributing to the aggravation of rheumatoid arthritis in transplanted nude mice. The CD153 and PD-1 double positivity rate of LIP-treated helper T cells induced by TCR stimulation (Day 2; 51.87 ± 11.49% versus 70.06 ± 8.78%, ***p = 0.00088, *n* = 10 mice/group) and the OPN production (Day 2; 2.24 ± 0.40 ng/mL versus 3.21 ± 0.66 ng/mL, *p = 0.012, *n* = 6 mice/group) were approximately 1.4-fold higher in helper T cells from the LIP group than in those from the CTL group (**Figures 2H**, **3A**). The arthritis index in nude mice injected with helper T cells from the LIP groups was also 1.4-fold higher (**Figure 7**). Furthermore, GO and KEGG enrichment analyses identified numerous senescence-related terms in the LIP group (**Figures 5C–F**). While these findings suggest that immunosenescence in the LIP group contributes to the exacerbation of arthritis in nude mice, direct scientific evidence remains insufficient to confirm that LIP-induced helper T-cell senescence alone initiates or drive arthritis in this model. Future investigations are warranted to distinguish the specific roles of senescent T cells from those of memory T cells. It should be noted, however, that the transferred T cells were unlikely to have undergone re-sensitization in the recipient nude mice, as these mice did not receive silk ligation.

Our findings demonstrate that severe periodontal inflammation, characterized by alveolar bone resorption, induces a “senescence-primed” status in the helper T cells. These cells may enter the systemic circulation and exacerbate rheumatoid arthritis, a key systemic condition within the framework of periodontal medicine. This discovery could facilitate the development of pre-symptomatic interventions, risk assessment models, and novel therapeutic strategies for periodontal-related systemic diseases. Furthermore, by providing robust scientific evidence that “oral health is integral to systemic health,” these results may catalyze a shift in healthcare paradigms, promoting the social recognition of routine oral care as a critical preventive measure for chronic diseases such as diabetes and rheumatoid arthritis.

In summary, this study elucidates a novel cellular mechanism–driven by T-cell senescence–linking periodontitis to pathogenesis of rheumatoid arthritis. These insights not only contribute to tooth preservation but also suggest that the managing oral inflammation may serve as a “pivotal switch” to mitigate systemic aging and inflammaging.

## Data Availability

The original data of RNA-seq in the paper has been uploaded into NCBI database with the identifier GSE318778.
