# Supplementary material for: Ligature-induced periodontitis in mice potentially accelerates CD4+ T-cell senescence and exacerbates rheumatoid arthritis
Source: Front Immunol. 2026 May 26;17:1806138. doi: 10.3389/fimmu.2026.1806138 (PMC13246652; doi:10.3389/fimmu.2026.1806138)
Supplement: Supplementary file 1 [file DataSheet1.docx]

**
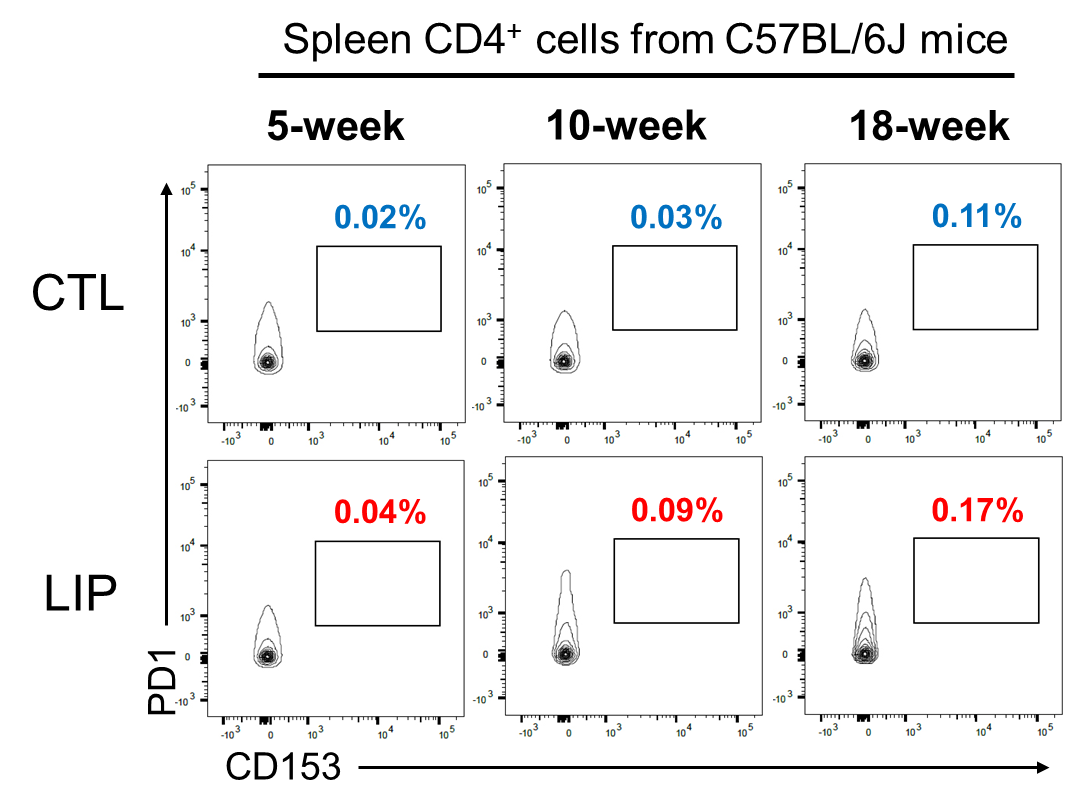
Supplementary Figure 1**

**Supplementary Figure 1.** PD-1^+^CD153^+^CD4^+^ T cells were also rarely present in the spleen of C57BL/6J mice of any age (5–18 weeks). All plots were gated on live CD4^+^ T cells. Representative plots display the percentages of PD-1^+^ and CD153^+^ cells in the spleen of the LIP and CTL groups. Similar results were obtained in five independent experiments.
