# Supplementary material for: Ligature-induced periodontitis in mice potentially accelerates CD4+ T-cell senescence and exacerbates rheumatoid arthritis
Source: Front Immunol. 2026 May 26;17:1806138. doi: 10.3389/fimmu.2026.1806138 (PMC13246652; doi:10.3389/fimmu.2026.1806138)
Supplement: Supplementary file 2 [file DataSheet2.docx]

**
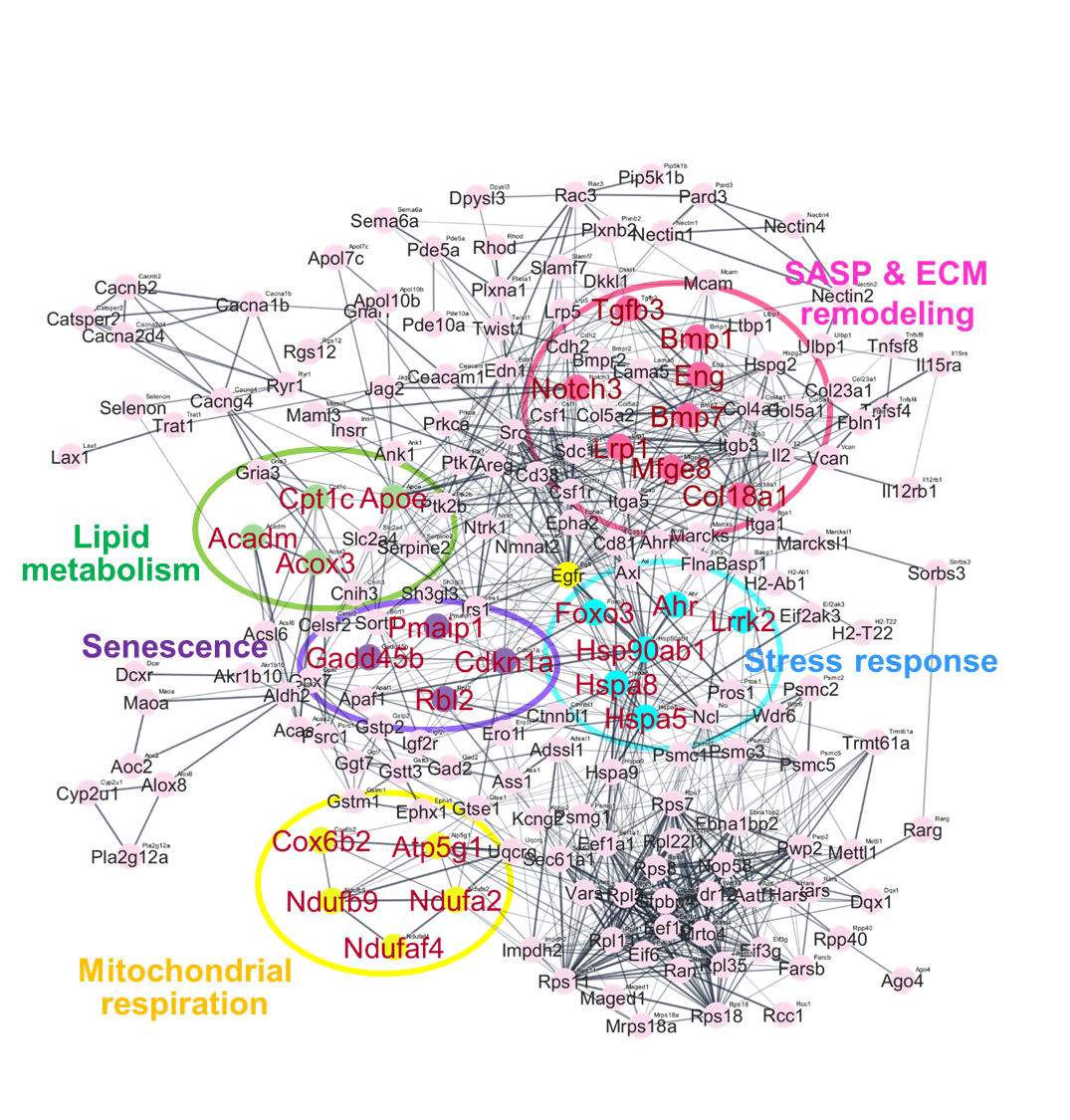
Supplementary Figure 2**

**Supplementary Figure 2.** Functional clustering of the PPI network in CD4^+^ T cells on day 2. The PPI network was constructed based on DEGs (*p* < 0.05). Genes are categorized into five functional modules indicated by colored nodes and frames: purple (senescence), red (SASP and extracellular matrix remodeling), green (lipid metabolism), blue (stress response), and yellow (mitochondrial respiration).
