# Supplementary material for: Ligature-induced periodontitis in mice potentially accelerates CD4+ T-cell senescence and exacerbates rheumatoid arthritis
Source: Front Immunol. 2026 May 26;17:1806138. doi: 10.3389/fimmu.2026.1806138 (PMC13246652; doi:10.3389/fimmu.2026.1806138)
Supplement: Supplementary file 3 [file Table1.docx]

**Supplementary Table 1**

Primer sequences used for quantitative real-time PCR

| **Gene** | **Forward primer (5′–3′)** | **Reverse primer (5′–3′)** |
| --- | --- | --- |
| ***Cx3cr1*** | GAGTATGACGATTCTGCTGAGG | CAGACCGAACGTGAAGACGAG |
| ***18S rRNA*** | GCTTAATTTGACTCAACACGGGA | AGCTATCAATCTGTCAATCCTGTC |
| ***Ifnβ*** | ACTGCCTTTGCCATCCAAGA | CACTGTCTGCTGGTGGAGTT |
| ***Mmp9*** | CTCTGCTGCCCCTTACCAG | ATAGCGGTACAAGTATGCCTCTG |
| ***Ccr6*** | ATGCGGTCAACTTTAACTGTGG | CCCGGAAAGATTTGGTTGCCT |
| ***Il1β*** | GAAGAAGAGCCCATCCTCTG | TCATCTCGGAGCCTGTAGTG |
